# Dietary Glycemic Load and Glycemic Index and Risk of Coronary Heart Disease and Stroke in Dutch Men and Women: The EPIC-MORGEN Study

**DOI:** 10.1371/journal.pone.0025955

**Published:** 2011-10-05

**Authors:** Koert N. J. Burger, Joline W. J. Beulens, Jolanda M. A. Boer, Annemieke M. W. Spijkerman, Daphne L. van der A

**Affiliations:** 1 National Institute for Public Health and the Environment, Bilthoven, The Netherlands; 2 Julius Center for Health Sciences and Primary Care, University Medical Center Utrecht, Utrecht, The Netherlands; Medizinische Hochschule Hannover, Germany

## Abstract

**Background:**

The associations of glycemic load (GL) and glycemic index (GI) with the risk of cardiovascular diseases (CVD) are not well-established, particularly in men, and may be modified by gender.

**Objective:**

To assess whether high dietary GL and GI increase the risk of CVD in men and women.

**Methods:**

A large prospective cohort study (EPIC-MORGEN) was conducted within the general Dutch population among 8,855 men and 10,753 women, aged 21–64 years at baseline (1993–1997) and free of diabetes and CVD. Dietary intake was assessed with a validated food-frequency questionnaire and GI and GL were calculated using Foster-Powell's international table of GI. Information on morbidity and mortality was obtained through linkage with national registries. Cox proportional hazards analysis was performed to estimate hazard ratios (HRs) for incident coronary heart disease (CHD) and stroke, while adjusting for age, CVD risk factors, and dietary factors.

**Results:**

During a mean follow-up of 11.9 years, 581 CHD cases and 120 stroke cases occurred among men, and 300 CHD cases and 109 stroke cases occurred among women. In men, GL was associated with an increased CHD risk (adjusted HR per SD increase, 1.17 [95% CI, 1.02–1.35]), while no significant association was found in women (1.09 [0.89–1.33]). GI was not associated with CHD risk in both genders, while it was associated with increased stroke risk in men (1.27 [1.02–1.58]) but not in women (0.96 [0.75–1.22]). Similarly, total carbohydrate intake and starch intake were associated with a higher CHD risk in men (1.23 [1.04–1.46]; and 1.24 [1.07–1.45]), but not in women.

**Conclusion:**

Among men, high GL and GI, and high carbohydrate and starch intake, were associated with increased risk of CVD.

## Introduction

Cardiovascular diseases (CVD) are a major cause of death worldwide. In Europe 54% of women and 43% of men die of CVD [1]. Hyperglycemia, insulin resistance, and associated disorders of lipid metabolism (hyperlipidemia) are key determinants of CVD, and these determinants are in turn influenced by diet [2], [3]. High carbohydrate diets may promote hyperglycemia, and can raise fasting triacylglycerol and reduce HDL levels [4]–[6], which may eventually increase the risk of CVD. Postprandial hyperglycemia is emerging as an independent and clinically significant risk factor for CVD [7], [8]. Therefore, high carbohydrate diets may increase the risk of CVD.

However, dietary carbohydrates produce different glycemic responses not only depending on their chemical structure, but also on particle size, fiber content, and food processing [9]. These differences between carbohydrate-containing foods can be expressed in the glycemic index (GI) which is a measure of the postprandial glucose response [10], and can be considered an indicator of the quality of food carbohydrates. Glycemic load (GL) is calculated by multiplying the GI of a food with its carbohydrate content and represents both quality and quantity of carbohydrates.

The Nurses' Health Study provided first evidence for an increased risk of CVD in women consuming high GL or high GI diets, especially among those who are overweight [11], [12]. These results were confirmed in a study of Dutch women who consumed a diet with a more modest glycemic load [13]. However, one large and two smaller studies among men did not reveal any association between dietary GL or GI and CVD risk [14]–[16], suggesting effect modification by sex. Women have a more favorable lipid profile with lower fasting LDL and higher HDL levels, and a less pronounced postprandial lipid response as a result of higher adipose LPL activity[17]. In view of these and other sex-specific differences (which also depend on age), the effects of high GI and GL on CVD risk may differ between men and women[2], [18].

So far, only two studies were able to make a direct comparison between men and women. In an Italian cohort study, both a high dietary GL and carbohydrate intake from high-GI foods were associated with an increased risk of CHD among women but not among men [19]. Similarly, in a Japanese cohort study, dietary GI was positively associated with fatal stroke risk among women only [20]. Therefore, the objective of the present study is to assess whether high dietary GL, GI, and high intakes of carbohydrate (subtypes), are associated with increased risk of CHD as well as stroke, in a large cohort of Dutch men and women consuming a modest glycemic load diet, and whether this association differs between men and women.

## Methods

### Ethics Statement

The study complied with the Declaration of Helsinki and was approved by the Medical Ethical Committee of TNO Nutrition and Food Research. All participants gave written informed consent prior to inclusion.

### Population

The EPIC-MORGEN cohort consists of 22,654 men and women aged 20–65 years selected from random samples of the Dutch population in 3 towns (Amsterdam, Doetinchem, and Maastricht) in the Netherlands between 1993 and 1997 [21], [22]. All participants gave informed consent prior to inclusion. Participants underwent a medical examination and filled in a general and food frequency questionnaire (FFQ). After exclusion of those who gave no consent to linkage with disease registries (n = 2097), who had a history of type 2 diabetes (n = 194) or CVD (n = 526), had missing nutritional data (n = 62), and/or were ranked in the top or bottom 0.5% of the ratio of reported energy intake over estimated basal metabolic rate (BMR; n = 210), a total of 19,608 participants were eligible for analysis (cumulative exclusion, n = 3,046).

### Baseline Measurements

The general questionnaire contained questions on demographics, presence of and risk factors for chronic diseases. Physical activity was assessed by a questionnaire, and categorized using the validated Cambridge Physical Activity Index [23]. Physical activity data were not available in the correct format for the first year of the EPIC-MORGEN study. To reduce bias, missing CPAI scores (25% of total) were imputed by single linear regression modeling [SPSS MVA procedure]. Missing data were almost absent (<0.6%) for other potential confounders and intermediates. Educational level was based on the highest level achieved and categorized into low, middle and high [22]. Anthropometric and blood pressure measurements were performed as described previously [22]. Hypertension was defined as present when one or more of the following criteria were met: diastolic blood pressure ≥90 mm Hg, systolic blood pressure ≥140 mm Hg, self-reported antihypertensive medication use, or self-reported presence of hypertension. Hypercholesterolemia was defined as a self-reported physician diagnosis. Smoking was categorized into never, former, and current smoker. Menopause was defined as the absence of menstrual periods for at least a year (including surgical menopause). Oral contraceptive (OC) use and postmenopausal hormone replacement therapy (HRT) use was defined as ever versus never. At baseline, all participants donated a non-fasting blood sample. Plasma total cholesterol and HDL cholesterol levels were determined using standardized enzymatic methods.

### Dietary Information

Daily nutritional intake was obtained at baseline from a FFQ containing questions on the usual frequency of consumption of 79 main food groups during the year preceding enrollment. This FFQ has been validated against twelve 24-h recalls [24]–[26]. Pearson correlations were 0.63 for GL and GI (men and women), 0.74 (men) and 0.76 (women) for carbohydrate, and 0.61 (men) and 0.74 (women) for fiber. The GI of foods was obtained from international tables using glucose as the reference [27], [28]. We calculated daily GI by multiplying the GI value of each food item with its carbohydrate content and frequency of consumption, and dividing the sum of these values over all food items by the total amount of carbohydrate consumed [29]. Daily GL was calculated in the same manner but without dividing by the total amount of carbohydrate consumed [30].

### Morbidity and Mortality Follow-up

Data on morbidity were obtained from the Dutch Centre for Health Care Information, which holds a register of hospital discharge diagnoses from all general and university hospitals in the Netherlands starting from 1990. The database was linked to the cohort on the basis of a validated probabilistic method [31]. The principal diagnosis, coded according to the Ninth Revision of the International Classification of Diseases (ICD-9-CM), was used to define the morbidity endpoints. Information on vital status was available through linkage with the municipal administration registries, and causes of death were obtained from Statistics Netherlands. Causes of death were coded according to ICD-9 for deaths until 1996 and ICD-10 thereafter. CHD (ICD-9-CM 410 to 414; ICD-10-CM I20 to I25) and stroke (ICD-9-CM 430 to 434, 436; ICD-10-CM I60 to I66) were the main end points of interest, combining fatal events (primary and secondary cause of death) and non-fatal events. In addition, we differentiated between ischemic stroke (ICD-9-CM 433, 434; ICD-10-CM I63, I65) and hemorrhagic stroke (ICD-9-CM 430 to 432; ICD-10-CM I60 to I62).

### Statistical Analysis

GL, GI, and intakes of nutrients were adjusted for total energy intake by means of the regression residual method [32]. Person-years of follow-up were calculated from the date of return of the questionnaires to the date of CHD or stroke, emigration, death or January 1 2008, whichever came first. Selected confounding variables were incorporated into multivariate Cox proportional hazard models stratified by sex. First, HRs were adjusted for age (continuous; model M1). Next, CVD risk factors were added: smoking, packyears (continuous), education, BMI (continuous), physical activity, hypertension, and OC use (in women; model M2). In the third model, total energy (continuous), and energy-adjusted intake of alcohol (≤10 g/day, >10–25 g/day, >25–50 g/day, >50 g/day), vitamin C, fiber, saturated, monounsaturated, and polyunsaturated fat (continuous) were added. Models for GI were also adjusted for carbohydrates and protein (continuous), while models for sugar and starch were mutually adjusted. Finally, we evaluated the effect of potential intermediate factors, by including total cholesterol and HDL-cholesterol (continuous; model M4). Nonlinear associations were explored by inclusion of quadratic terms, and were all non-significant (*P*>0.11). Interactions with sex, BMI, age, and menopausal status were tested using the likelihood ratio test. In a sensitivity analysis we excluded energy under-reporters (energy intake-to-BMR ratio of less than 1.14) [33]. The proportional hazard assumption was checked visually using log-minus-log plots with no deviations detected. Data were analyzed with SAS (version 9.2; SAS Institute Inc., Cary, NC) for Windows.

## Results

Daily mean (± SD) energy-adjusted dietary GL was lower in men (121.8±21.0) than in women (125.2±19.8), while dietary GI was similar (Table 1). The main contributors to GI were bread (18%), milk products (18%), alcoholic and non-alcoholic beverages (16%), potatoes (16%), and fruit (13%). Daily GL was largely determined by consumption of bread (35%), potatoes (14%) and sweets (13%). On average, men consumed more alcohol and less vitamin C, were more often diagnosed with hypercholesterolemia, and had lower HDL-cholesterol levels. Men and women in the highest quartile of dietary GL consumed more carbohydrates, sugar and starch, less protein and fat, more fiber and vitamin C, and less alcohol than did those in the lowest quartile. They were also younger and had a lower BMI, they were less highly educated and smoked less, and, especially men, were less likely to be hypertensive (data not shown).

**Table 1:** Baseline Characteristics* of the EPIC-MORGEN cohort According to Sex.

	Men	Women
Variable		
N (n,[%])	8855 [45.2]	10753 [54.8]
Glycemic Load (g/d)	121.8 (21.0)	125.2 (19.8)
Glycemic Index	55.4 (4.1)	55.2 (3.6)
Age (yrs)	43.0 (11.0)	42.1 (11.3)
BMI (kg/m2)	25.4 (3.5)	24.7 (4.2)
Physical Activity (%)		
Inactive	12.0	9.5
Moderately Inactive	31.3	31.8
Moderately Active	28.3	30.1
Active	28.5	28.7
Education (%)		
Low	32.6	35.3
Middle	40.1	43.7
High	27.4	21.1
Smoking (%)		
Never	31.3	37.8
Former	30.9	27.3
Current	37.9	34.9
Hypertension (%)	30.5	31.8
Hypercholesterolemia (%)	18.7	8.5
Total cholesterol (mmol/l)	5.3 (1.1)	5.3 (1.1)
HDL-cholesterol (mmol/l)	1.19 (0.30)	1.51 (0.37)
Menopausal status (% post)		23.4
OC use (%)		84.7
HRT use (%)		12.0
Dietary intake†		
Energy (kcal/d)	2603 (666)	1984 (496)
Carbohydrates (g/d)	221.9 (30.1)	226.2 (29.9)
Sugar (g/d)	105.7 (29.1)	111.7 (29.6)
Starch (g/d)	115.4 (22.7)	114.4 (23.1)
Protein (g/d)	74.6 (10.2)	74.4 (10.9)
Total Fat (g/d)	75.6 (10.5)	78.2 (10.9)
Polyunsaturated Fat (g/d)	14.8 (3.7)	15.2 (3.7)
Monounsaturated Fat (g/d)	29.1 (4.8)	30.1 (5.1)
Saturated Fat (g/d)	31.0 (5.2)	32.4 (5.5)
Dietary Fiber (g/d)	22.8 (5.0)	22.8 (4.6)
Alcohol (g/d)	10.4 (13.3)	9.0 (15.4)
Dietary Vitamin C (mg/d)	92.4 (38.2)	108.8 (43.5)

* Mean (SD); †all nutritional variables were adjusted for total energy intake, except energy. BMI  =  body mass index; OC =  oral contraceptives; HRT  =  hormone replacement therapy.

During 233,697 person-years of follow-up, 581 CHD cases and 120 cases of stroke occurred among men, whereas 300 CHD cases and 109 stroke cases occurred among women.

GL was positively associated with the risk of CHD in men, with an HR of 1.12 (95% CI: 1.03–1.21) per SD increase, after adjusting for established CVD risk factors (model M2, Table 2), while no association was found in women (HR: 1.01; CI: 0.90–1.14). Including nutritional factors (model M3) slightly augmented the risk in men (HR: 1.17; CI: 1.02–1.35) and women, but the risk in women remained non-significant (HR: 1.09; CI: 0.89–1.33). Dietary GL was not associated with an increased stroke risk in men (HR: 1.22; CI: 0.89–1.66) or women (HR: 0.91; CI: 0.65–1.27), although the effect size in men was similar to that observed for CHD risk. After adjustment for CVD risk factors and nutrients, GI was related to stroke risk in men only (HR: 1.27; CI: 1.02–1.58; versus HR in women: 0.96; CI: 0.75–1.22), while no association was observed with CHD risk, neither in men nor women.

**Table 2:** Glycemic Load, Glycemic Index, and the Risks of Coronary Heart Disease and Stroke Among 8,855 Men and 10,753 Women*.

		CHD		Stroke	
		Men	Women	Men	Women
	Cases	581	300	120	109
GL	M1: age	1.01 (0.93–1.09)	0.97 (0.86–1.08)	0.99 (0.83–1.17)	0.94 (0.78–1.14)
	M2: M1 + CVD risk factors†	1.12 (1.03–1.21)	1.01 (0.90–1.14)	1.06 (0.89–1.27)	0.97 (0.80–1.18)
	M3: M2 + nutrients‡	1.17 (1.02–1.35)	1.09 (0.89–1.33)	1.22 (0.89–1.66)	0.91 (0.65–1.27)
	M4: M3 + intermediates§	1.14 (0.99–1.32)	1.05 (0.86–1.28)	1.23 (0.90–1.69)	0.90 (0.65–1.26)
GI	M1: age	1.05 (0.97–1.13)	1.08 (0.96–1.22)	1.10 (0.92–1.30)	1.07 (0.88–1.30)
	M2: M1 + CVD risk factors†	1.09 (1.01–1.17)	1.03 (0.92–1.16)	1.12 (0.95–1.32)	1.02 (0.84–1.24)
	M3: M2 + nutrients‡ ∥	1.03 (0.93–1.14)	1.08 (0.93–1.25)	1.27 (1.02–1.58)	0.96 (0.75–1.22)
	M4: M3 + intermediates§∥	1.02 (0.92–1.13)	1.07 (0.92–1.24)	1.27 (1.02–1.58)	0.95 (0.75–1.22)

* Adjusted Hazard Ratios (with 95% CI) per SD of GL (20.5) and GI (3.9).

† Adjusted for age, smoking (never, past, current), packyears, education (low, middle, high), BMI, physical activity (inactive, moderately inactive, moderately active, active), hypertension (yes, no), and OC use (ever, never).

‡ Additional adjustment for total energy, and energy-adjusted nutrients: alcohol (≤10, 10–25, 25–50, >50 g/day), vitamin C, dietary fiber, and saturated, monounsaturated, and polyunsaturated fat. §Model 3, additionally adjusted for plasma total cholesterol, and HDL-cholesterol.

∥ Models M3 and M4 for GI were also adjusted for energy-adjusted carbohydrate and protein intake. Among the 229 stroke cases, 115 cases of ischemic stroke (68 among men), and 69 (25 among men) cases of hemorrhagic stroke were recorded. GL correlated strongly with carbohydrate intake (Pearson r>0.91), while GI did not (r<0.35).

CHD  =  coronary heart disease; GL  =  dietary glycemic load; GI  =  dietary glycemic index; M  =  model; HDL  =  high-density lipoprotein.

Total carbohydrate intake and starch intake were positively associated with CHD risk in men (Table 3), with HRs per SD increase of 1.23 (CI: 1.04–1.46) and 1.24 (CI: 1.07–1.45), respectively, but not in women. Sugar intake was associated with a slightly higher, although non-significant, risk of CHD in men. Inclusion of total and HDL cholesterol as potential intermediates in the model (model M4), attenuated the HRs for CHD in men (HRs were reduced 10–50%) while not reducing the HRs for stroke (Table 2 and 3).

**Table 3:** Total Carbohydrate, Sugar, and Starch, and the Risks of Coronary Heart Disease and Stroke Among 8,855 Men and 10,753 Women*.

		CHD		Stroke	
		Men	Women	Men	Women
	Cases	581	300	120	109
Carbohydrates	M1: age	0.99 (0.92–1.08)	0.93 (0.83–1.04)	0.94 (0.79–1.12)	0.90 (0.74–1.08)
	M2: M1 + CVD risk factors†	1.12 (1.03–1.21)	1.00 (0.89–1.12)	1.01 (0.84–1.21)	0.94 (0.78–1.14)
	M3: M2 + nutrients‡	1.23 (1.04–1.46)	1.04 (0.82–1.33)	1.01 (0.70–1.45)	0.91 (0.61–1.35)
	M4: M3 + intermediates§	1.20 (1.02–1.43)	1.00 (0.79–1.28)	1.02 (0.70–1.47)	0.90 (0.60–1.34)
Sugar	M1: age	0.96 (0.89–1.05)	1.01 (0.90–1.14)	0.96 (0.80–1.15)	0.93 (0.77–1.12)
	M2: M1 + CVD risk factors†	1.03 (0.95–1.12)	1.08 (0.97–1.21)	0.99 (0.83–1.19)	0.96 (0.80–1.16)
	M3: M2 + nutrients‡∥	1.17 (0.99–1.38)	1.10 (0.86–1.41)	1.00 (0.70–1.44)	0.96 (0.65–1.44)
	M4: M3 + intermediates§∥	1.15 (0.97–1.36)	1.05 (0.82–1.35)	1.01 (0.70–1.46)	0.95 (0.63–1.42)
Starch	M1: age	1.03 (0.95–1.12)	0.89 (0.79–1.00)	0.97 (0.81–1.16)	0.96 (0.79–1.16)
	M2: M1 + CVD risk factors†	1.09 (1.01–1.19)	0.90 (0.80–1.01)	1.02 (0.86–1.23)	0.99 (0.82–1.19)
	M3: M2 + nutrients‡∥	1.24 (1.07–1.45)	0.94 (0.76–1.17)	1.07 (0.76–1.50)	0.88 (0.62–1.25)
	M4: M3 + intermediates§∥	1.22 (1.04–1.42)	0.92 (0.74–1.14)	1.07 (0.76–1.51)	0.87 (0.61–1.24)

*Adjusted Hazard Ratios (with 95% CI) per SD of carbohydrates (30.1), sugar (29.5), and starch (22.9).

† Adjusted for age, smoking (never, past, current), packyears, education (low, middle, high), BMI, physical activity (inactive, moderately inactive, moderately active, active), hypertension (yes, no), and OC use (ever, never).

‡ Additional adjustment for total energy, and energy-adjusted nutrients: alcohol (≤10, 10–25, 25–50, >50 g/day), vitamin C, dietary fiber, and saturated, monounsaturated. §Model M3, additionally adjusted for plasma total cholesterol and HDL-cholesterol. ∥Models M3 and M4 for sugar and starch were mutually adjusted.

Separating stroke subtypes, both GL and GI were positively associated with risk of ischemic stroke as well as hemorrhagic stroke in men, but the association was only statistically significant for GI and ischemic stroke risk (HR: 1.34; CI: 1.01, 1.80). No association between GL or GI and stroke subtypes was observed in women. However, the low number of cases (69 in total, 25 among men) precludes an accurate analysis of hemorrhagic stroke risk.

All interactions of GL or GI with sex, BMI (below and above 25 kg/m^2^), age (median split), or menopausal status were not statistically significant. Only the interaction between GI and age for men was borderline significant (p = 0.07). Subgroup analysis by age (median 43.1 years) showed a stronger association of GI with CHD risk in the younger men (1.27; CI: 0.98–1.64) than in older men (0.98; CI: 0.88–1.10). Similar results were found for GL with HRs of 1.45 (CI: 1.01–2.06) and 1.14 (CI: 0.98–1.33; interaction p-value 0.55) respectively. The opposite was observed among women with a negative, non-significant, association of GL and GI with CHD risk in the younger women (0.80; CI: 0.47–1.38 and 0.89; CI: 0.61–1.30, respectively), and a positive, non-significant, association of GL and GI with CHD risk in the older age group (1.13; CI: 0.91–1.40 and 1.12; CI: 0.95–1.31, respectively).

Sensitivity analyses, replacing BMI by waist-hip-ratio or waist circumference did not appreciably affect the results. Associations did not change after adjustment for menopausal status or HRT use, removing OC use from the multivariate model or after exclusion of people with prevalent cancer (n = 492). The exclusion of CVD cases occurring in the first two years of follow-up, or of energy under-reporters (n = 4267), augmented the effects of GL on CHD risk in men (HR: 1.24; CI: 1.06–1.44; and 1.31; CI: 1.11–1.54) but not in women. Finally, restriction of the analysis to fatal outcome, confirmed the results showing a positive association of GL with CHD in men only (HR: 1.79; CI: 1.23–2.60).

## Discussion

Our main finding is that in this Dutch cohort consuming a modest GL diet, a high dietary GL and GI, and high total carbohydrate and starch intake, were associated with an increased CVD risk in men. In men, GI was associated with an increased stroke risk, while GL, carbohydrate and starch intake were associated with an increased CHD risk. Among women, no significant associations were observed.

Apart from a recent Swedish cohort study showing no significant associations of GL and GI with CVD in women [34], most studies have suggested an association of dietary GI and GL with CVD risk for women and not for men [11]–[16], [19], [20]. Our results suggest the opposite with positive associations for GI and GL among men, but not women, although it should be noted that the interactions of GI and GL with sex were not statistically significant. These discrepancies may be explained by differences between the study populations. First, differences in diet and dietary contributors to GI and GL between the study populations may contribute to these results. Higher carbohydrate consumption and GL are observed in the American, Italian and Japanese studies [11], [12], [16], [19], [20] and particular foods like pasta and rice contribute more strongly to GL in these studies than ours. However, dietary differences are unlikely to fully explain our results because studies in Dutch and Swedish cohorts with similar GL and contributors to GL also show results opposite to those in the current study [13]–[15].

Second, there are differences in the general characteristics and risk factors for chronic disease between the study populations. CVD risk among women may be influenced by OC and HRT use, and modified by menopausal status. In addition, the strongest associations of GL and GI with CVD risk were generally observed among overweight women [11]–[13], [16]. However, we could not detect an interaction with BMI, and adjustment for menopausal status, OC and HRT use did not influence our results. Finally, participants in our study were 20–66 years old (average 43 years) at baseline, considerably younger than in the other studies where average age is above 50 years. In a subgroup analysis comparing older and younger men, we observed a stronger association of GL and GI with CHD risk in the younger age group (average age 34 years) than in the older age group (average age 52 years), while our results suggest the reverse among women. Our results on the older age group are in reasonable agreement with the results found for similarly aged men and women in earlier studies. Thus, although the interaction with age did not reach statistical significance, it could potentially explain our results in comparison with previous studies. Moreover, the finding that younger men may be more sensitive to high GL and GI is an important message given the prevalence of obesity in adolescents and the role of nutrients contributing to high glycemic load in current dietary behavior in younger generations.

Our results are the first to show that dietary GI and GL are associated with an increased risk of CVD among men. Only in a small Finnish cohort study, a positive association of GL and GI with myocardial infarction risk among men was observed, but these associations were only significant among overweight or physically less active men [35]. In addition, a recent prospective cohort study indicated that replacing dietary saturated fatty acids with high-GI carbohydrates is associated with a higher risk of myocardial infarction particularly among men [36]. Altogether, our findings and the aforementioned studies suggest that high dietary GI and GL also increase risk of CVD among men. More observational studies are required to replicate our findings and to come to a final conclusion on the associations of dietary GI and GL with CVD risk among men and women.

In men, dietary GL increased CHD risk, while dietary GI increased stroke risk. However, the effect size of the association of dietary GL with stroke risk was similar to that of GI, but did not reach statistical significance (Table 2). So far, positive associations of GL or GI with stroke risk have only been observed among women, and a high stroke risk was either associated with GL [12], [13] or GI [20]. The slightly different associations of GI and GL with stroke could to some extent be explained by different associations for stroke subtypes. Two previous studies [12], [14] showed that a high dietary GL and carbohydrate intake were particularly associated with an increased risk of hemorrhagic stroke, but not ischemic stroke. Associations of dietary GI were similar for ischemic and hemorrhagic stroke. These studies suggest that a high carbohydrate intake and thus a high GL is predominantly associated with risk of hemorrhagic stroke. Since the vast majority of stroke cases are ischemic strokes, this may have attenuated the association of GL with stroke. Our data suggest that in men, the GI component of GL is responsible for the (non-significant) positive association of GL with stroke risk, while the carbohydrate component of GL appears to be responsible for the increased CHD risk. Up to now, associations of carbohydrate intake and CVD risk have not been reported for men. Contradictory results were obtained for women, with either no associations found [11], [13], [20], or positive associations [12], [19]. Our data suggest that total carbohydrate, starch, and GL, are equally strong predictors for CHD risk in men, whereas only GL and GI are predictors for stroke risk.

Randomized trials have shown that low-GI and low-GL diets affect plasma concentrations of LDL-cholesterol, HDL-cholesterol, total cholesterol, triglycerides and markers of inflammation and thrombosis, as well as insulin resistance, in ways that would be expected to reduce CVD risk [37]–[42]. The importance of lipid intermediates in determining CVD risk was also reflected in our analyses showing that the association between carbohydrate determinants and CHD risk was reduced by including plasma total cholesterol and HDL-cholesterol in the multivariate models. In contrast, associations with stroke risk were not reduced. Associations of CVD risk factors, such as hyperlipidemia and hypertension, with CHD and stroke risk have been shown to differ [43]–[45], and may be explained by etiological differences between CHD and stroke.

A strength of our study is its prospective design and large sample size. Residual confounding can not be excluded, but is made less likely by the large number of risk factors that we adjusted for. Misclassification of dietary exposure is a valid concern in studies that rely on self-report. Moreover, the FFQ was not specifically designed to measure dietary GL and GI. However, the Dutch EPIC FFQ has been validated showing good agreement with 24-h recalls for most food groups as well as for dietary GL and GI [24]–[26]. A previous study showed that underreporting influenced associations of dietary GI and GL with risk of diabetes [46]. Although, a broad exclusion of potential energy-underreporters did augment the effects of GL and GI on CVD risk in men, it did not result in a positive association of GL or GI with CVD risk in women. There has recently been some criticism with respect to the reliability and individual variability of GI [47]. Overall, GI appears to be a valid predictor of the glycemic response, also to mixed meals [48], [49]. Even though not every food with a low GI may be equally beneficial, GI represents a useful functional property that can help guide dietary choices that should also take total available and unavailable carbohydrate consumption into account [50].

To our knowledge, this is the first study to show that, also among men, dietary GL and GI may be associated with an increased CVD risk, but these findings need to be further replicated. Dietary GL, and carbohydrate and starch intake were associated with increased CHD risk, while dietary GI was associated with increased stroke risk in men. No associations were observed for women. Also considering earlier studies carried out in more aged study populations, dietary GL and GI emerge as potentially important determinants of CVD risk for both men and women. Notably, increments of 1 SD in dietary GL, GI, and carbohydrate were shown to be achievable in practice[51]. Therefore, reducing dietary GL and GI should be part of the nutritional advice for a healthy lifestyle.

## References

[1] Rayner M, Allender S, Scarborough P, British Heart Foundation Health Promotion Research Group (2009). Cardiovascular disease in europe.. Eur J Cardiovasc Prev Rehabil.

[2] Hoekstra T, Beulens JW, van der Schouw YT (2009). Cardiovascular disease prevention in women: Impact of dietary interventions.. Maturitas.

[3] Levitan EB, Cook NR, Stampfer MJ, Ridker PM, Rexrode KM (2008). Dietary glycemic index, dietary glycemic load, blood lipids, and C-reactive protein.. Metabolism.

[4] Frost G, Leeds AA, Dore CJ, Madeiros S, Brading S (1999). Glycaemic index as a determinant of serum HDL-cholesterol concentration.. Lancet.

[5] Liu S, Manson JE, Stampfer MJ, Holmes MD, Hu FB (2001). Dietary glycemic load assessed by food-frequency questionnaire in relation to plasma high-density-lipoprotein cholesterol and fasting plasma triacylglycerols in postmenopausal women.. Am J Clin Nutr.

[6] McKeown NM, Meigs JB, Liu S, Rogers G, Yoshida M (2009). Dietary carbohydrates and cardiovascular disease risk factors in the framingham offspring cohort.. J Am Coll Nutr.

[7] Gerich JE (2003). Clinical significance, pathogenesis, and management of postprandial hyperglycemia.. Arch Intern Med.

[8] Aryangat AV, Gerich JE (2010). Type 2 diabetes: Postprandial hyperglycemia and increased cardiovascular risk.. Vasc Health Risk Manag.

[9] Aston LM (2006). Glycaemic index and metabolic disease risk.. Proc Nutr Soc.

[10] Jenkins DJ, Wolever TM, Taylor RH, Barker H, Fielden H (1981). Glycemic index of foods: A physiological basis for carbohydrate exchange.. Am J Clin Nutr.

[11] Liu S, Willett WC, Stampfer MJ, Hu FB, Franz M (2000). A prospective study of dietary glycemic load, carbohydrate intake, and risk of coronary heart disease in US women.. Am J Clin Nutr.

[12] Oh K, Hu FB, Cho E, Rexrode KM, Stampfer MJ (2005). Carbohydrate intake, glycemic index, glycemic load, and dietary fiber in relation to risk of stroke in women.. Am J Epidemiol.

[13] Beulens JW, de Bruijne LM, Stolk RP, Peeters PH, Bots ML (2007). High dietary glycemic load and glycemic index increase risk of cardiovascular disease among middle-aged women: A population-based follow-up study.. J Am Coll Cardiol.

[14] Levitan EB, Mittleman MA, Hakansson N, Wolk A (2007). Dietary glycemic index, dietary glycemic load, and cardiovascular disease in middle-aged and older swedish men.. Am J Clin Nutr.

[15] van Dam RM, Visscher AW, Feskens EJ, Verhoef P, Kromhout D (2000). Dietary glycemic index in relation to metabolic risk factors and incidence of coronary heart disease: The zutphen elderly study.. Eur J Clin Nutr.

[16] Tavani A, Bosetti C, Negri E, Augustin LS, Jenkins DJ (2003). Carbohydrates, dietary glycaemic load and glycaemic index, and risk of acute myocardial infarction.. Heart.

[17] Lopez-Miranda J, Williams C, Lairon D (2007). Dietary, physiological, genetic and pathological influences on postprandial lipid metabolism.. Br J Nutr.

[18] Knopp RH, Paramsothy P, Retzlaff BM, Fish B, Walden C (2006). Sex differences in lipoprotein metabolism and dietary response: Basis in hormonal differences and implications for cardiovascular disease.. Curr Cardiol Rep.

[19] Sieri S, Krogh V, Berrino F, Evangelista A, Agnoli C (2010). Dietary glycemic load and index and risk of coronary heart disease in a large italian cohort: The EPICOR study.. Arch Intern Med.

[20] Oba S, Nagata C, Nakamura K, Fujii K, Kawachi T (2010). Dietary glycemic index, glycemic load, and intake of carbohydrate and rice in relation to risk of mortality from stroke and its subtypes in japanese men and women.. Metabolism.

[21] Riboli E, Hunt KJ, Slimani N, Ferrari P, Norat T (2002). European prospective investigation into cancer and nutrition (EPIC): Study populations and data collection.. Public Health Nutr.

[22] Verschuren WM, Blokstra A, Picavet HS, Smit HA (2008). Cohort profile: The doetinchem cohort study.. Int J Epidemiol.

[23] Wareham NJ, Jakes RW, Rennie KL, Schuit J, Mitchell J (2003). Validity and repeatability of a simple index derived from the short physical activity questionnaire used in the european prospective investigation into cancer and nutrition (EPIC) study.. Public Health Nutr.

[24] Ocké MC, Bueno-de-Mesquita HB, Goddijn HE, Jansen A, Pols MA (1997). The dutch EPIC food frequency questionnaire. I. description of the questionnaire, and relative validity and reproducibility for food groups.. Int J Epidemiol.

[25] Ocké MC, Bueno-de-Mesquita HB, Pols MA, Smit HA, van Staveren WA (1997). The dutch EPIC food frequency questionnaire. II. relative validity and reproducibility for nutrients.. Int J Epidemiol.

[26] Du H, van der A DL, van Bakel MM, Verberne LD, Ocké M (2009). Reproducibility and relative validity of dietary glycaemic index and glycaemic load assessed by the food-frequency questionnaire used in the dutch cohorts of the european prospective investigation into cancer and nutrition.. Br J Nutr.

[27] Foster-Powell K, Holt SH, Brand-Miller JC (2002). International table of glycemic index and glycemic load values: 2002.. Am J Clin Nutr.

[28] Atkinson FS, Foster-Powell K, Brand-Miller JC (2008). International tables of glycemic index and glycemic load values: 2008.. Diabetes Care.

[29] Wolever TM, Nguyen PM, Chiasson JL, Hunt JA, Josse RG (1994). Determinants of diet glycemic index calculated retrospectively from diet records of 342 individuals with non-insulin-dependent diabetes mellitus.. Am J Clin Nutr.

[30] Salmeron J, Manson JE, Stampfer MJ, Colditz GA, Wing AL (1997). Dietary fiber, glycemic load, and risk of non-insulin-dependent diabetes mellitus in women.. JAMA.

[31] Herings RM, Bakker A, Stricker BH, Nap G (1992). Pharmaco-morbidity linkage: A feasibility study comparing morbidity in two pharmacy based exposure cohorts.. J Epidemiol Community Health.

[32] Willett WC, Howe GR, Kushi LH (1997). Adjustment for total energy intake in epidemiologic studies.. Am J Clin Nutr.

[33] Goldberg GR, Black AE, Jebb SA, Cole TJ, Murgatroyd PR (1991). Critical evaluation of energy intake data using fundamental principles of energy physiology: 1. derivation of cut-off limits to identify under-recording.. Eur J Clin Nutr.

[34] Levitan EB, Mittleman MA, Wolk A (2010). Dietary glycaemic index, dietary glycaemic load and incidence of myocardial infarction in women.. Br J Nutr.

[35] Mursu J, Virtanen JK, Rissanen TH, Tuomainen TP, Nykänen I (2011). Glycemic index, glycemic load, and the risk of acute myocardial infarction in finnish men: The kuopio ischaemic heart disease risk factor study.. Nutr Metab Cardiovasc Dis.

[36] Jakobsen MU, Dethlefsen C, Joensen AM, Stegger J, Tjønneland A (2010). Intake of carbohydrates compared with intake of saturated fatty acids and risk of myocardial infarction: Importance of the glycemic index.. Am J Clin Nutr.

[37] Rizkalla SW, Taghrid L, Laromiguiere M, Huet D, Boillot J (2004). Improved plasma glucose control, whole-body glucose utilization, and lipid profile on a low-glycemic index diet in type 2 diabetic men: A randomized controlled trial.. Diabetes Care.

[38] McMillan-Price J, Petocz P, Atkinson F, O'neill K, Samman S (2006). Comparison of 4 diets of varying glycemic load on weight loss and cardiovascular risk reduction in overweight and obese young adults: A randomized controlled trial.. Arch Intern Med.

[39] Maki KC, Rains TM, Kaden VN, Raneri KR, Davidson MH (2007). Effects of a reduced-glycemic-load diet on body weight, body composition, and cardiovascular disease risk markers in overweight and obese adults.. Am J Clin Nutr.

[40] Pereira MA, Swain J, Goldfine AB, Rifai N, Ludwig DS (2004). Effects of a low-glycemic load diet on resting energy expenditure and heart disease risk factors during weight loss.. JAMA.

[41] Ebbeling CB, Leidig MM, Sinclair KB, Seger-Shippee LG, Feldman HA (2005). Effects of an ad libitum low-glycemic load diet on cardiovascular disease risk factors in obese young adults.. Am J Clin Nutr.

[42] Dickinson S, Hancock DP, Petocz P, Ceriello A, Brand-Miller J (2008). High-glycemic index carbohydrate increases nuclear factor-kappaB activation in mononuclear cells of young, lean healthy subjects.. Am J Clin Nutr.

[43] Chrysant SG, Chrysant GS (2010). Effectiveness of lowering blood pressure to prevent stroke versus to prevent coronary events.. Am J Cardiol.

[44] Schürks M, Glynn RJ, Rist PM, Tzourio C, Kurth T (2010). Effects of vitamin E on stroke subtypes: Meta-analysis of randomised controlled trials.. BMJ.

[45] Leppälä JM, Virtamo J, Fogelholm R, Albanes D, Heinonen OP (1999). Different risk factors for different stroke subtypes: Association of blood pressure, cholesterol, and antioxidants.. Stroke.

[46] Sluijs I, van der Schouw YT, van der A DL, Spijkerman AM, Hu FB (2010). Carbohydrate quantity and quality and risk of type 2 diabetes in the european prospective investigation into cancer and nutrition-netherlands (EPIC-NL) study.. Am J Clin Nutr.

[47] Williams SM, Venn BJ, Perry T, Brown R, Wallace A (2008). Another approach to estimating the reliability of glycaemic index.. Br J Nutr.

[48] Wolever TM (1997). The glycemic index: Flogging a dead horse?. Diabetes Care.

[49] Wolever TM (2010). Another approach to estimating the reliability of the glycaemic index: A different interpretation.. Br J Nutr.

[50] Howlett J, Ashwell M (2008). Glycemic response and health: Summary of a workshop.. Am J Clin Nutr.

[51] Ebbeling CB, Leidig MM, Feldman HA, Lovesky MM, Ludwig DS (2007). Effects of a low-glycemic load vs low-fat diet in obese young adults: A randomized trial.. JAMA.

